# In Vivo Safety Characterization of Injectable Amidated TEMPO-Oxidized Cellulose Nanofiber Hydrogel Vaccine Formulations in Farmed Atlantic Salmon (*Salmo salar* L.)

**DOI:** 10.3390/vaccines14040313

**Published:** 2026-03-31

**Authors:** Sarah M. Turner, Michael Mason, Jacob A. Holbrook, Jeongwhui Hong, Inga F. Sidor, Deborah A. Bouchard

**Affiliations:** 1 Cooperative Extension and Aquaculture Research Institute, University of Maine, Orono, ME 04469, USA; deborah.bouchard@maine.edu; 2 Department of Biomedical Engineering, University of Maine, Orono, ME 04469, USA; michael.mason@maine.edu (M.M.); jacob.holbrook@maine.edu (J.A.H.); 3 Department of Aquaculture, Chonnam National University, Yeosu 59626, Republic of Korea; jhong@chonnam.ac.kr; 4 New Hampshire Veterinary Diagnostic Laboratory, University of New Hampshire, Durham, NH 03824, USA; inga.sidor@unh.edu

**Keywords:** biopolymers, cellulose nanomaterials, hydrogels, vaccines, adjuvants, Atlantic salmon, aquaculture, shear-thinning

## Abstract

**Background:** Disease outbreaks remain a major constraint on aquaculture production, making vaccination essential for disease management in farmed fish. However, injectable oil-adjuvanted vaccines can be costly and may induce adverse inflammatory reactions and welfare concerns, motivating investigations into alternative injectable adjuvant materials. **Methods:** We conducted an *in vivo* safety evaluation of shear-thinning, amidated TEMPO-oxidized cellulose nanofiber (TO-CNF) hydrogels formulated with an inactivated *Vibrio anguillarum* bacterin. Formulations were administered intraperitoneally to Atlantic salmon (*Salmo salar* L.) using a common garden design with cohabitated treatment groups across triplicate tanks. Fish were monitored and sampled at pre-injection baseline and at 300-, and 600-degree days post-injection. Safety endpoints included mortality, macroscopic and histopathological outcomes, and growth evaluated relative to sham controls, unmodified TO-CNF, and a commercial oil-adjuvanted vaccine. **Results:** Amidated TO-CNF formulations were associated with increased mortality (up to 16–18% in higher reagent-loading groups) compared to commercial oil-adjuvanted vaccine, material, and sham controls. Affected fish exhibited adverse outcomes, including adhesions, proliferative lesions, ascites, edema, hemorrhage, and secondary opportunistic infections. In contrast, controls showed minimal mortality and pathology. Growth and immune response endpoints were variable and did not demonstrate consistent treatment-associated effects. Physicochemical analyses indicated differences in formulation stability and qualitative compositional differences across modification levels, but these were not quantified nor linked to specific causal mechanisms in this study. **Conclusions:** The amidated TO-CNF formulations tested here were associated with formulation-dependent safety risks under the conditions evaluated and are not yet suitable as injectable vaccine adjuvants in Atlantic salmon. These findings define important safety constraints for this material class and highlight the need for improved modification and purification strategies. More broadly, this work underscores the importance of establishing *in vivo* safety boundaries prior to efficacy optimization for emerging biomaterial-based vaccine adjuvants.

## 1. Introduction

Disease outbreaks remain a major constraint on global aquaculture production, resulting in substantial economic losses and persistent challenges to animal welfare and sustainability [1,2]. Due to the inherently high infectious pressure in intensive aquaculture, injectable vaccination has become a widely used approach for preventive disease management in finfish production [3,4]. This strategy delivers small, concentrated doses of antigen(s) to stimulate the adaptive immune system, supporting both humoral and cell-mediated immune responses in fish [5,6,7,8]. However, currently used oil-adjuvanted vaccines are frequently associated with adverse outcomes due to strong up-regulation of the innate immune response, including significant growth penalties and welfare issues such as visceral adhesions and internal organ damage [9,10,11,12,13,14,15]. Atlantic salmon (*Salmo salar* L.) are among the most extensively farmed finfish species and vaccination has contributed substantially to increased productivity by preventing disease [16,17,18]. However, salmon can also experience negative impacts attributed to vaccine-induced inflammatory responses [11,12,19]. Consequently, there remains a need to develop cost-effective vaccination strategies that elicit robust and durable immune protection while minimizing adverse welfare effects.

Biopolymer-based materials derived from natural sources have emerged as cost-effective candidates for vaccine applications due to their tunable physicochemical properties, biodegradability, and potential biocompatibility [20,21,22]. Among these materials, hydrogels have received attention as vaccine delivery systems because they can function as localized depots, enable sustained antigen release, and support the co-delivery of immunomodulatory compounds [23,24,25]. Despite theoretical advantages, the practical application of hydrogel-based vaccines has been limited in aquaculture with safety and *in vivo* tolerability poorly characterized in finfish. Highly crosslinked and/or stiff three-dimensional hydrogels often require surgical implantation or generate substantial mechanical trauma during administration [26], and in Atlantic salmon can elicit pronounced foreign body responses [27]. These constraints have restricted the translational utility of three-dimensional hydrogels in finfish vaccination and highlight a need for improved injectability.

Shear-thinning injectable biopolymer hydrogels represent a potential strategy to address these delivery challenges. These formulations transiently decrease in viscosity under shear stress (i.e., shear-thinning during injection) and recover their gel structure *in situ* through hydrogelation. Despite growing interest in shear-thinning hydrogels for biomedical and veterinary applications [28,29,30,31], *in vivo* exploration in aquatic species remains limited, especially in the context of commercially relevant vaccine delivery routes and adjuvants. However, the *in vivo* safety of such chemically modified, injectable hydrogel systems remain poorly defined in finfish.

Cellulose nanomaterials (CNM), including cellulose nanofibers (CNF), are abundant, renewable polysaccharides (biopolymers) at the nanoscale that have been widely studied for biomedical applications including drug delivery due to their high surface area, mechanical strength, and capacity for surface functionalization [32,33,34,35,36]. TEMPO-oxidized cellulose nanofibers (TO-CNF) introduce carboxyl groups along the cellulose backbone, enabling chemical modification and electrostatic interactions that can be leveraged to form hydrogel networks suitable for injectable biomedical and vaccine delivery applications [30,32,36]. Previous studies have demonstrated that unmodified CNMs exhibit favorable safety and biocompatibility in a range of *in vitro* and *in vivo* mammalian models; however, species-specific responses in finfish remain poorly characterized [37,38,39]. The chemical modification of TO-CNFs through amidation has been used to introduce hydrophobic side chains to produce shear-thinning hydrogel matrices with tailored mechanical properties and injectability for drug delivery and tissue engineering applications in terrestrial systems, including cellulose-based materials [40,41,42]. Although amidation chemistry has been studied to tailor CNM mechanical properties in drug delivery applications, its biological consequences following intraperitoneal administration in finfish remain largely unexplored.

The introduction of reactive reagents and hydrophobic moieties through amidation chemistry raises important questions regarding *in vivo* safety in aquatic animals which may be sensitive to formulation-related factors such as residual chemicals or material-induced inflammation. To date, the authors are unaware of studies that have systematically examined the biological consequences of chemically modified CNM hydrogels following intraperitoneal administration in finfish. As a result, the safety and design constraints governing the use of amidated TO-CNF hydrogels as injectable vaccine adjuvants remain poorly defined. Defining safety boundaries is a necessary initial step before efficacy-focused development of cellulose-based hydrogel vaccine platforms for aquaculture.

In this study, the safety of unmodified TO-CNF intraperitoneally injected into Atlantic salmon observed during pilot examination (unpublished) prompted our research team to conduct *in vivo* characterization of shear-thinning, amidated TO-CNF hydrogels formulated with an inactivated *Vibrio anguillarum* bacterin and administered intraperitoneally in Atlantic salmon. The objective of this study was to evaluate the *in vivo* safety, pathological outcomes, and biological responses associated with intraperitoneal administration of amidated TO-CNF hydrogels compared to currently used commercially oil-adjuvanted vaccines in Atlantic salmon. Immunological responses were assessed to first characterize immune activation before protective efficacy. Physicochemical characterization was conducted in parallel to relate formulation properties to observed biological outcomes. This work aims to define critical safety limitations associated with injectable amidated TO-CNF hydrogels prior to efficacy-focused development, thereby informing the design of safer biopolymer-based vaccine adjuvants for aquaculture. This study provides an *in vivo* evaluation of chemically amidated cellulose nanofiber hydrogels administered by intraperitoneal injection in finfish and characterizes the safety constraints for the tested formulations.

## 2. Materials and Methods

### 2.1. Preparation of Vaccine Using CNM Matrices as an Adjuvant

#### 2.1.1. Bacterin Production

*Vibrio anguillarum* isolate VE-2021-0143 was obtained from the University of Maine Aquatic Animal Health Laboratory (AAHL)’s frozen bacterial stock culture collection. The inactivated *V. anguillarum* bacterin acted as the antigen and was prepared as previously reported and stored at 4 °C until use [27].

#### 2.1.2. Amidated TEMPO-Oxidized CNF Production

A 1.1 wt% (*w*/*w*) TEMPO-oxidized CNF slurry (manufacturer-reported average fiber length ≈ 1 µm; diameter ≈ 20 nm; Process Development Center, University of Maine, USA) was used as stock material and diluted with deionized (DI) water to a final concentration of 0.5 wt% (*w*/*w*). The slurry was stirred at 600–700 rpm and equilibrated at 50 °C in a water bath prior to further processing. Next, 1-ethyl-3-(3-dimethylaminopropyl) carbodiimide hydrochloride (EDC, 95%; Fisher Scientific, Rockford, IL, USA) and N-Hydroxysuccinimide (NHS, 98%; Fisher Scientific, Rockford, IL, USA) were dissolved in 3.0 mL of DI water. Reagent masses were normalized to a fixed TO-CNF dry mass per formulation, such that increasing ‘1×’ and ‘2×’ conditions reflect increased reagent loading relative to constant TO-CNF content. In the 1× ALL TO-CNF formulation, all reagents were loaded at 1× (1.0088 g EDC, 0.7267 g NHS, and 1.131 g ODA). In the 2× EDC formulation, reagent loading was doubled to 2.02 g EDC and 1.45638 g NHS while maintaining constant ODA and TO-CNF content. In the 2× ODA formulation, EDC/NHS and TO-CNF were held constant (1.00869 g and 0.72680 g, respectively).

The dissolved EDC/NHS solution was added to the 0.5 wt% (*w*/*w*) TEMPO-oxidized CNF suspension at 1× and 2× concentrations and stirred until uniformly mixed. The pH was adjusted to 5.5–6.0 using 1 M sodium hydroxide (NaOH; Fisher Scientific, Nazareth, PA, USA) and allowed to stir for an additional 30 min. The octadecylamine (ODA, 90%; Fisher Scientific, Fair Lawn, NJ, USA) was manually ground with a mortar and pestle to a fine powder and combined with dimethylformamide (DMF, 99.8%; Acros Organics, Fair Lawn, NJ, USA). For the 1× ALL TO-CNF formulation, 1.131 g ODA was dissolved in 32.5 mL DMF. In the 2× EDC formulation, 1.157 g ODA was dissolved in 33 mL DMF. In the 2× ODA formulation, ODA loading was doubled to 2.26383 g and dissolved in 65 mL DMF while EDC, NHS, and TO-CNF content remained constant. The mixtures were sonicated in continuous mode at an amplitude of 75% for 2 min 30 s (Fisherbrand™ Model 505 Sonic Dismembrator, Pittsburg, PA, USA).

The pH was adjusted to 7.5–8.0 using 1 M HCl (Fisher Scientific, Nazareth, PA, USA) and 1 M NaOH (Fisher Scientific, Nazareth, PA, USA) and stirred continuously for 24 h. The TO-CNF formulations were washed three times by centrifugation (7000× *g* rpm for 5 min; fixed-angle rotor) with reconstitution of the pellet in DI water, then once in ethanol pH adjusted to 3.0 with 0.1 N HCl, and then twice more in DI water. Fourier transform infrared spectroscopy was performed on supernatants collected from the wash steps during production and final formulations to qualitatively assess the removal of residual reaction components. Each sample was then placed into 3500 Da dialysis tubing (Fisher Scientific, Rockford, IL, USA) for 24 h to further reduce any potential residual components. After dialysis, the samples were mixed at a ratio of 1:10 amidated TO-CNF: *Vibrio anguillarum* antigen and stored at 4 °C until use. Each formulation was prepared as a single batch and subdivided for *in vivo* administration and concurrent physicochemical characterization. Batch-to-batch variability was not assessed, as the study was designed to evaluate formulation-level safety under defined preparation conditions rather than manufacturing reproducibility. A summary of formulation composition, relative reagent loading, and intended functional differences is provided in Table 1 to clarify experimental design and treatment comparisons. Relative reagent loading (1× vs. 2×) refers to proportional increases in coupling or modification reagents during synthesis and does not reflect changes in TO-CNF concentration per formulation.

An additional 2× ALL formulation was prepared by doubling ODA, DMF, EDC, and NHS relative to the 1× ALL formulation while maintaining constant TO-CNF content. This formulation was produced using the same sonication, washing, and dialysis. It was included for *in vitro* physicochemical characterization only to assess the combined effects of increased reagent loading on hydrogel composition, structure, and rheology and was not advanced to *in vivo* evaluation.

### 2.2. In Vivo Evaluation of Amidated TO-CNF in Atlantic Salmon

#### 2.2.1. In Vivo Study

Five hundred unvaccinated Atlantic salmon parr weighing approximately 50 g were obtained from Cooke Aquaculture’s hatchery (Bingham, ME, USA) and transferred to the University of Maine Cooperative Extension Diagnostic and Research Laboratory (Orono, ME, USA). Of the total 500 fish received, 360 fish were randomly allocated to the three main 490 L experimental tanks (120 fish per tank across three replicate tanks; average stocking density of 12 kg/m^3^), with the remaining fish assigned to unvaccinated sentinel control tanks or later used in separate DPBS + Bacterin control tanks and allowed to acclimate for 14 days.

The study system consisted of a freshwater well water partial flow-through and recirculating system at 12 ± 0.5 °C with a mechanical bead filter, bio-filter, UV disinfection, and eight 490 L tanks. Each tank was supplied with 2 L min^−1^ oxygenated water to maintain a dissolved oxygen level of 8.5 ± 2.0 mg/L. Water and fish parameters—which included temperature (°C), dissolved oxygen (mg/L), feed observation, fish appearance, mortalities, total ammonia (mg/L), and total nitrite (mg/L) levels—were documented daily. Tanks were also siphoned daily to remove particulates. The salmon were fed a commercial diet (Bio-Oregon, Westbrook, ME, USA) twice daily at a feed rate of 1.5% body weight per day.

Twenty Atlantic salmon parr per six formulations in triplicate tanks (120 fish per tank) were anesthetized in 100 mg/L MS-222 buffered with sodium bicarbonate and elastomer tagged with an identifying color corresponding to treatment groups before being intraperitoneally injected with a 1 mL syringe and 22-gauge needle (Becton-Dickinson, Canaan, CT, USA). Injection was slightly posterior to the pelvic fin and perpendicular to the ventral surface on the left side. Formulations were coded numerically during administration, and the injection operator was blinded to treatment identity. Elastomer tag colors corresponding to treatment groups were also blinded throughout the study and downstream analysis. Fish were injected with a standard dose volume of 100 μL of one of six treatment formulations. These included: (1) DPBS only (sham negative control); (2) unmodified TO-CNF + Bacterin (material control); (3) sonicated 1× ALL TO-CNF + Bacterin; (4) 2× ODA TO-CNF + Bacterin; (5) 2× EDC TO-CNF + Bacterin; and (6) a commercial oil-adjuvanted vaccine (Montanide™ ISA 763 A VG adjuvant, Richmond, VA, USA) formulated with bacterin (positive control) (Table 1). The commercial oil-adjuvanted vaccine was included as a practical benchmark for a field-relevant injectable formulation with established safety performance. Each treatment consisted of 20 fish per tank co-habitated in three replicate tanks in a common garden design to control for tank-level environmental effects. This design resulted in 60 fish per treatment across the three replicate tanks.

Unvaccinated sentinel fish (*n* = 120) were held in separate duplicate tanks within the system. Following injection, fish were immediately returned to the respective tanks for recovery. A DPBS + Bacterin group (*n* = 20) was injected and added to the study in a separate tank four days later to confirm that observed adverse events were not attributable to the bacterin itself. This control was introduced post hoc, housed separately, sampled on the appropriate number of days post-injection, and should therefore be interpreted with caution relative to cohabitated treatment groups.

Sampling was performed in a blinded manner on 30 Atlantic salmon pre-injection baseline and at 300- and 600-degree days (12 °C) post-injection by euthanizing fish with a lethal dose of MS-222 supplemented with sodium bicarbonate. Collected serum and head kidney samples were stored at −80 °C until processed. Furthermore, predefined humane endpoints were implemented to minimize animal suffering throughout the study. Humane endpoints were defined as fish reaching moribund condition or exhibiting clinical signs of distress, including respiratory gasping, pronounced lethargy, loss of equilibrium, and/or development of severe lesions. These fish were promptly euthanized as previously described and in accordance with approved University of Maine Institutional Animal Care and Use Committee (IACUC Protocol #: A2023-02-01). Mortalities, therefore, included both spontaneous deaths and fish euthanized upon reaching humane endpoints. Fish that died prior to sampling were excluded from downstream analyses; sample sizes therefore reflect surviving subsampled individuals at each time point.

#### 2.2.2. Observed Biometrics

Fish were monitored twice daily for feeding vigor throughout the study. Biometric data of weight (g) and fork length (mm) were measured to calculate Fulton’s condition factor (*K*) and mean specific growth rate (SGR or %*G*) at pre-injection baseline and at 300- and 600-degree days post-injection to assess growth effects [43,44].

#### 2.2.3. External and Internal Gross Examination

Gross necropsy was performed on all fish to assess adverse responses associated with administration and dispersion of amidated TO-CNF hydrogels. Complete external and internal gross examinations were performed at pre-injection (baseline), 300- and 600-degree days post-injection, and on all mortalities. External examination evaluated injection healing and presence of external lesions including protruding proliferative tissue. Internal visceral and peritoneal adhesions, melanization, fibrosis, granuloma formation, and vaccine residue were evaluated and scored based on a modified Spielberg scale [43]. Any other observations deviating from normal were described. Histology tissues were collected. Head kidney samples were collected and stored at −80 °C for real-time qPCR analysis.

#### 2.2.4. Histopathology

At the conclusion of the *in vivo* study (600-degree days post-injection), tissue samples were collected from 3 fish per treatment group per triplicate tank (*n* = 54), along with ten unvaccinated sentinel fish for a total of 64 samples. Samples from each fish were fixed in 35 mL of 10% neutral buffered formalin (Fisher Scientific, Fair Lawn, NJ, USA). Tissues were not collected for histological analysis from the DPBS + Bacterin control group. Tissues included approximately 1 cm^2^ of body wall including skeletal muscle layers bordering the injection site, the pyloric caeca, liver, a section of digestive tract (stomach and/or intestine), and spleen. Tissues were submitted to the New Hampshire Veterinary Diagnostic Laboratory (NHVDL, Durham, NH, USA) for histopathologic processing and evaluation. Tissues were routinely processed, embedded in paraffin, sectioned at 5 μm thickness, mounted on charged slides, and stained with hematoxylin and eosin. Tissue samples were blinded to treatment groups and scored on the appearance of inflammation, fibrosis, and granulation tissue according to the rubric previously presented in Turner et al., 2024 [27], and the presence or absence of injected foreign material (vaccine) was noted.

### 2.3. Exploratory Examination of In Vivo Immune Markers in Atlantic Salmon

#### 2.3.1. Real-Time qPCR Assay

RNA isolation, reverse transcription and real-time qPCR assays for immune genes were performed as previously described [45]. A subset of three fish per treatment per tank replicate was analyzed to balance representation across tanks while minimizing additional animal use. Head kidney was collected from Atlantic salmon at 300- and 600-degree days post-vaccination and stored at −80 °C until use. Total RNA was isolated from the head kidney of Atlantic salmon using TRIzol^®^ reagent (Life Technologies, Carlsbad, CA, USA). The samples were placed in 2 mL tubes containing 1 mL of TRIzol^®^ and homogenized in the Omni Bead Ruptor Elite (Kennesaw, GA, USA), then 200 µL of chloroform was added with vigorous mixing before being incubated at 4 °C for 10 min. The samples were then centrifuged at 12,000× *g* at 4 °C for 15 min. The supernatants containing RNA (400 µL) were transferred to new 1.5 mL tubes, and the RNA was precipitated by adding 400 µL of isopropanol, which were then mixed gently before incubating overnight at −80 °C. RNA pellets were collected by centrifuging at 12,000× *g* at 4 °C for 10 min, washed with 1 mL of cold 75% ethanol. The supernatants were discarded, and RNA pellets were air-dried for 5 min before they were resuspended in 100 µL nuclease-free water. The purity and quantity of extracted RNA were assessed by NanoDrop™ Spectrophotometer (Thermo Scientific™, Wilmington, DE, USA) (260/230 and 260/280 ratio ≥ 1.8). The cDNA of samples was synthesized using PrimeScript™ RT reagent kit (Takara Bio, San Jose, CA, USA), following the manufacturer’s instructions. Real-time quantitative PCR was carried out on QuantStudio 3 (Applied Biosystems, Carlsbad, CA, USA) in 20 µL total volume reactions and 500 nmol primers according to the protocol provided by the manufacturer. PCR cycling conditions for all genes consisted of an initial denaturation at 95 °C for 30 s, followed by 40 cycles of 95 °C for 5 s and 60 °C for 34 s. Melt curve analysis was performed with a dissociation step at 95 °C for 30 s, 60 °C for 30 s, and a gradual increase to 95 °C to verify that a single PCR product was produced. The relative expression of genes involved in immune response (*IgM*, *IgT* and *IgD*) were determined using primers designed from Atlantic salmon sequences in the NCBI database. Primer sequences for genes are given in Table 2. The reference gene, *β-actin*, was used to normalize the expression levels of the target genes and was selected based on its widespread use in teleost immune studies for relative gene expression analysis. All primers of the target and reference genes were synthesized by the Integrated DNA Technologies (IDT, Morrisville, NC, USA). The amplification efficiencies of the target and reference genes were quantified according to the specific gene standard curves generated from 10-fold serial dilutions. After verifying that the primers were amplified with 100% efficiency, the relative expression results were analyzed using the 2^−ΔΔCt^ method with expression normalized to *β-actin* and calculated relative to sham-injected DPBS controls at the corresponding time point [46].

#### 2.3.2. Serological Antigen Specific Antibody Response by Indirect ELISA

An indirect enzyme-linked immunosorbent assay (ELISA) was used to quantify *Vibrio anguillarum*-specific IgM antibody titers in serum from Atlantic salmon vaccinated with amidated TO-CNF formulations or a commercial oil-adjuvanted vaccine at 300- and 600-degree days post-vaccination. These analyses were intended to assess immune activation rather than protective efficacy. Whole-cell inactivated *V. anguillarum* bacterin was prepared as previously described and used as the coating antigen (1.0 × 10^8^ CFU) [27]. The bacterin was centrifuged at 3500× *g* for 15 min. The cell pellet was resuspended in an equal volume of DPBS and stored at 4 °C until ready for use. Serum samples from a subset of three fish per treatment, per tank replicate (*n* = 9 per treatment) were analyzed to balance statistical representation across tanks while minimizing additional animal use. Negative control serum consisted of pooled sentinel unvaccinated Atlantic salmon serum, and assays were performed using consistent conditions across plates to minimize inter-assay variability. Serial two-fold dilutions of serum were performed in duplicate wells. ELISAs were performed according to manufacturer protocols (Aquatic Diagnostics Ltd., Oban, Scotland, UK), using whole-cell coating antigen, with incubations performed for 24 h at 4 °C between each coating step. The primary antibody was mouse anti-salmonid IgM (Aquatic Diagnostics, Oban, Scotland, UK), and the secondary antibody was goat anti-mouse IgG–HRP (Sigma-Aldrich, St. Louis, MO, USA). Plates were developed using 100 µL per well 1-Step™ Ultra TMB ELISA Substrate Solution (3,3′,5,5′ tetramethylbenzidine; Fisher Scientific, Fair Lawn, NJ, USA) and the reaction was stopped after 10 min by adding 50 µL per well of 2 M sulfuric acid (Fisher Scientific, Hanover, PA, USA). Plates were mixed and the absorbance was recorded at 450 nm using a spectrophotometer (Agilent, BioTek Synergy™, Winooski, VT, USA). Antibody titer calculation and statistical comparisons are described below in Section 2.5.

### 2.4. Examining In Vitro Characteristics of Amidated TO-CNF Hydrogel Formulations

#### 2.4.1. Mechanical Properties: Rheology

The rheological characterization of changes in viscosity of the formulations under force was examined at the University of Maine’s Biomedical Engineering Mason Laboratory (Orono, ME, USA). A parallel-plate rheometer (DHR-3, TA Instruments, Lindon, UT, USA) equipped with a 4 mm radius upper plate was used to approximate flow behavior of the material under shear conditions relevant to syringe injection conditions by determining the flow and deformation (mechanical) characteristics of the amidated TO-CNF formulations at room temperature (25 °C). Shear rate (*γ*, s^−1^), shear stress (τ, Pa), and apparent viscosity (η, Pa·s) were recorded. The formulations examined were unmodified TO-CNF, 1× ALL TO-CNF with and without sonication, 2× ODA TO-CNF with and without sonication, 2× EDC TO-CNF with and without sonication, and 2× ALL TO-CNF with and without sonication. Each formulation was tested in triplicate. The lower plate remained fixed. A 0.5 mL aliquot of material was added. The height was lowered to 1050 μm. Excess material was removed to reduce additional drag forces and subsequent error in measurements. The height was then reduced to 1000 μm before rotational torque was applied. The upper plate continuously rotated with an applied force or shear stress. As the rotational speed (shear rate, *γ*, s^−1^) increased in a step manner, the longer that shear rate was applied to equilibrate the material. Viscosity (η, Pa·s) was quantified. From the quantified shear rate and viscosity, log-log plots were produced to describe the relative flow behavior of the formulations under shear conditions relevant to injection. These measurements were intended for comparative assessment and do not represent a direct replication of *in vivo* injection conditions.

#### 2.4.2. Structural Properties: Scanning Electron Microscopy

Scanning electron microscopy (SEM; Zeiss NVision 40 FIB/SEM, Oberkochen, Germany) was performed at the University of Maine Electron Microscopy Laboratory (Orono, ME, USA) to visualize amidated TO-CNF samples. Sonication was incorporated during production of the 1× ALL TO-CNF hydrogels formulation to assess its effect on material homogeneity. Four formulations were examined for comparison by SEM: (1) 1× ALL TO-CNF, (2) sonicated 1× ALL TO-CNF, (3) sonicated 2× EDC TO-CNF, and (4) sonicated 2× ODA TO-CNF. Samples were flash frozen in liquid nitrogen and then freeze dried for 30 h to reduce the presence of ice templating within the formulations. The plate temperature was cycled through −34.4, −6.7, 4.4, 15.6 and 32.2 °C for 8, 10, 8, 3, and 3 h, respectively, under a constant vacuum of 0.1 Torr. After drying, the formulations were resubmerged in liquid nitrogen and fractured to create a clear cross section. The formulations were attached to SEM stubs using conductive epoxy for imaging. The formulations were metal coated by vacuum evaporating 6 nm of gold/palladium using a Denton DV-502 Rotary Evaporator (Moorestown, NJ, USA) and then sputter coated with an additional 2 nm of gold/palladium to ensure adequate metal coating. Each sample was imaged at 25×, 100×, 250×, and 1500× magnification.

#### 2.4.3. Chemical Properties: Fourier Transform Infrared Spectroscopy

FT-IR (ATR accessory-equipped Nicolet iS20, Fisher Scientific, Waltham, MA, USA) was used qualitatively to track disappearance/persistence of diagnostic peaks during formulation production and to analyze the chemical composition of the final various amidated TO-CNF formulations compared to unmodified TO-CNF. Samples were collected for FT-IR throughout production directly after the 24 h amidation, the first wash, and the third wash steps. This was performed to evaluate changes consistent with removal of residual or trace chemicals during the wash steps and to qualitatively assess spectral changes consistent with polymer modification in the 1× ALL TO-CNF formulation. The spectra were recorded on a diamond plate with a resolution of 4 cm^−1^ with 128 scans. The attenuated total reflection (ATR) auto-correction parameter was enabled for higher precision. The wavelength of the spectrum was set from 4000 to 400 cm^−1^. Prior to sample acquisition, the background was set to the same parameters as sample acquisition. Liquid and amidated TO-CNF samples (200 μL) were loaded onto the diamond plate and covered to limit evaporation during sampling. Solid samples fully covered the diamond plate in powdered form before the ATR compression arm was engaged. The absorbance spectra were separated for comparison by graphing manually with offset Y-axis values.

### 2.5. Calculations and Statistical Analysis

Sample sizes for the *in vivo* trial were determined by power analysis to detect differences in the modified Spielberg scale of tissue reaction and antibody response in Atlantic salmon between the different treatment groups using G Power Version 3.1 software (Heinrich Hein University, Düsseldorf, Germany) [48]. Degree day was calculated by multiplying the mean water temperature during the course of the study by the number of study days (*DD* = ((*T*_0_ + *T*_1_ + …)/no. of days) × no. of days) [49]. For adverse events, cumulative incidence (%) was calculated by the number of mortalities per treatment group divided by the number of total fish in the treatment group by 600-degree days. Cumulative censored (%) was calculated by the number of censored fish per treatment group divided by the number of total fish in the treatment group by 600-degree days. Fulton’s condition factor (*K*) was calculated using 100*WL*^−3^ where *W* is body weight (g) and *L* is fork length (cm) [43]. Mean SGR (%*G*) was calculated for each treatment by replicate tank using *G* (%) = ((Ln(*w_f_*) − Ln(*w_i_*)) × 100)/*t* where w_f_ is the mean weight (g) of the treatment within a replicate tank at 300- or 600-degree days, *w_i_* is the mean weight (g) of the fish at the baseline sample, and *t* is the number of true days at the time point [43].

Tank effects and tank × treatment interactions were evaluated statistically prior to primary analysis to ensure that treatment effects were not confounded by tank-level environmental variation and that statistical comparisons reflected treatment responses rather than system-level artifacts. No significant tank-related effects were detected (*p* > 0.05); therefore, data were pooled across replicate tanks for treatment-level analyses.

Normality of condition factor and SGR were assessed using Shapiro–Wilk tests. These endpoints were analyzed using ordinary two-way ANOVA, followed by Tukey’s HSD post hoc tests where appropriate. Mortality data were analyzed using Kaplan–Meier time-to-death analysis, with survival curves compared using the Gehan–Breslow–Wilcoxon test. Mortality included both spontaneous death and humane euthanasia; both were included in survival analysis. Moribund fish requiring humane euthanasia were identified based on predefined clinical observational criteria and recorded at the time of removal. All mortality events occurred prior to the first post-injection sampling time point; therefore, subsequent analyses (biometrics, pathology, ELISA, and qPCR) were conducted on surviving fish only. No missing data due to mortality were present within sampling time points. Mantel-Haenszel hazard ratios were calculated for each treatment group relative to the commercial oil-adjuvanted vaccine, which served as the referent due to its role as the current industry standard and practical benchmark for evaluating formulation safety. Groups with a *p* < 0.05 were considered statistically significant. Gross and microscopic lesions scores were evaluated using the modified Spielberg rubric and histopathology rubric, respectively, and compared between treatment groups at each timepoint using non-parametric Kruskal–Wallis ANOVA followed by Dunn’s post hoc tests where indicated.

Relative gene expression data were assessed for normality by Shapiro–Wilk tests and analyzed using ordinary two-way ANOVA with Dunnett’s post hoc test comparing treatments to the DPBS negative control. ELISA samples were run in duplicate and data were processed by calculating corrected mean OD_450nm_ values from duplicate wells. Assay validity was assessed using standard accepted intra- and inter-assay coefficients of variation, with target criteria of <10% and <15%, respectively. Endpoint titers were defined as the final doubling dilution exceeding the pooled negative control serum mean (derived from sentinel unvaccinated fish) + 3 standard deviations. Inter-assay variability (plate-to-plate) was controlled by including internal controls on each plate. Titers were compared between treatment groups at each timepoint using Kruskal–Wallis ANOVA.

Primary endpoints were predefined safety outcomes, including survival, mortality risk, and gross pathological responses following injection. Secondary physiological indicators of fish health, including specific growth rate (SGR) and condition factor, were analyzed to evaluate potential impacts of treatment on overall fish performance.

Secondary endpoints, including gene expression and antigen-specific antibody responses, were considered exploratory. Because multiple immune parameters were evaluated, increasing the potential for false positive findings, these analyses were interpreted as hypothesis-generating rather than confirmatory tests. Accordingly, statistical significance associated with these exploratory endpoints should be interpreted cautiously, and immune response data are presented primarily to provide biological context for the observed safety outcomes rather than definitive evidence of adjuvant efficacy.

*In vitro* measurements were intended for comparative and descriptive characterization of formulation behavior rather than hypothesis testing. Shear rate, shear stress, viscosity, power-law slope, and flow behavior index were calculated according to established methods [50]. Log-log plots of viscosity versus shear rate and shear stress versus shear rate were used to characterize flow behavior and calculate the flow behavior index (n). Integrated absorbance areas from FT-IR spectra were calculated for comparative analysis. Rheological calculations and figure generation were performed using Origin 2022b (OriginLab Corporation, Northampton, MA, USA).

All statistical analyses were performed using GraphPad Prism 9.5.1 (Boston, MA, USA) unless otherwise noted. Results are reported as mean ± standard error of the mean (SEM). For primary endpoints, *p* < 0.05 was considered statistically significant.

## 3. Results

### 3.1. Amidated TO-CNF Hydrogels Are Associated with Reagent-Loading-Dependent Adverse Outcomes in Atlantic Salmon

Primary safety endpoints included mortality, pathology, and survival, while growth and immune responses were evaluated as secondary or exploratory outcomes. A total of twenty-five mortalities occurred during the study period, with triplicate experimental tanks experiencing 6.7, 6.7, and 7.5% total mortality, respectively. Mantel-Haenszel hazard ratios (HR) with 95% confidence intervals (95% CI) were calculated to compare mortality risk among treatments. Mortality included both spontaneous death (17/25 events) and humane euthanasia of moribund fish (8/25 events; three events from 1× ALL and five events from the higher modification groups). Both were included in survival analyses, consistent with standard time-to-event methodology. All mortality events (spontaneous and humane endpoints) occurred prior to the 300-degree day sampling time point; therefore, all fish sampled at 300- and 600-degree days represent surviving individuals. Unvaccinated sentinel fish, sham-injected controls (DPBS Only and DPBS + Bacterin), and the unmodified TO-CNF + Bacterin groups exhibited lower mortality relative to the commercial oil-adjuvanted vaccine. Intraperitoneal vaccination with TO-CNF amidated using 1× reagent loading (1× ALL TO-CNF) showed increased mortality in Atlantic salmon; however, survival curves were not significantly different from the commercial oil-adjuvanted vaccine based on Gehan–Breslow–Wilcoxon analysis. Hazard ratio estimates for treatment groups with zero or very low mortality events produced wide confidence intervals. This imprecision reflects the limited number of observed mortality events rather than instability in the model itself, and these estimates should therefore be interpreted cautiously as indicative rather than precise measures of relative mortality risk. Higher reagent loading used to amidate TO-CNF was associated with significantly increased risk of mortality in 2× ODA TO-CNF and 2× EDC TO-CNF formulations compared to the commercial oil-adjuvanted vaccine. Because TO-CNF content was held constant across formulations, these differences are consistent with increasing amidation reagent loading rather than changes in polymer dose per injection. These results are summarized in Table 3.

**Figure 1:**
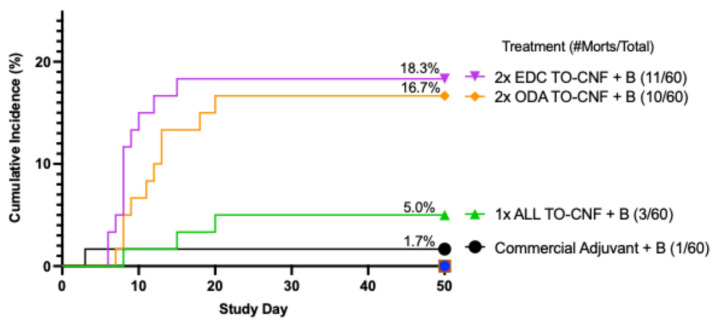
Kaplan–Meier curves displaying cumulative mortality incidence (%) for Atlantic salmon vaccinated with amidated TO-CNF treatments compared to positive commercial oil-adjuvanted vaccine during *in vivo* study. No mortalities (0% cumulative incidence) occurred in the sentinel, negative sham, antigen or material control fish during the course of the study as indicated by the colored symbols seen at 0% on study day 50. Each vertical step in the curve indicates one or more events (deaths) from time of vaccination (study day) over 600-degree days. The Gehan–Breslow–Wilcoxon test for comparing cumulative incidence of mortalities (%) across all treatment groups during the course of the study indicates significant differences are present (*p* < 0.0001; *n* = 60).

Kaplan–Meier time-to-event analysis compared the cumulative incidence of mortalities (%) across each treatment group during the course of the study with significant differences observed between treatments using a Gehan–Breslow–Wilcoxon analysis (*x*^2^ = 56.96, *p* < 0.0001) (Figure 1).

### 3.2. Fish Growth Metrics Were Largely Maintained over the Study Period

**Figure 2:**
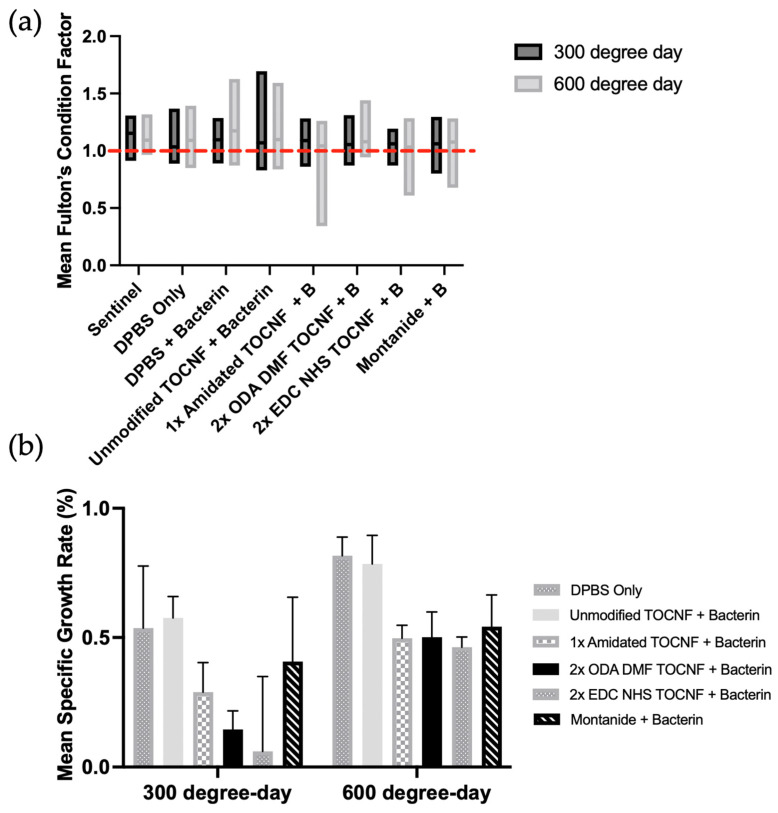
Growth metrics of Atlantic salmon monitored at 300- and 600-degree days post-injection. (**a**) Mean Fulton’s condition factor (*K*) of Atlantic salmon between formulations over the course of the trial. No significance between formulations (*p* = 0.7628) nor time (*p* = 0.8671). Dotted red line denotes *K* = 1.0. (**b**) Mean-specific growth rate (%) of Atlantic salmon vaccinated by treatment group at 300- and 600-degree days post-injection. Two-way ANOVA shows significant main effects of time point (*p* = 0.0057) and treatment (*p* = 0.0416) with no significant pairwise differences detected by post hoc analysis. Error bars represent standard error of the mean (*n* = 30).

Fish maintained vigorous feeding and normal behavior throughout the study, though transient reductions were observed in some high-reagent groups following injection. A two-way ordinary ANOVA analysis was conducted to evaluate the effect of vaccine formulation and sampling time point on Fulton’s condition factor (*K*) in intraperitoneally vaccinated Atlantic salmon parr. Sentinel unvaccinated fish and DPBS + Bacterin groups were not included in statistical analyses due to unequal sample sizes; however, their mean *K* values were comparable to those of the analyzed treatment groups and shown in Figure 2a. Analysis revealed no statistically significant interaction between formulation nor time point (*F*(5, 319) = 1.073, *p* = 0.3754) on *K*. Simple main effects analysis showed the formulations did not have a statistically significant effect on *K* (*F*(5, 319) = 0.5177, *p* = 0.7628) and time point did not have a statistically significant effect on *K* (*F*(1, 319) = 0.02805, *p* = 0.8671). Mean *K* was greater than 1.0 across all formulations at 300-degree days (*K*_M300_ = 1.073, *SD* = 0.141) and at 600-degree days (*K*_M600_ = 1.077, *SD* = 0.139) including in the sentinel fish (*K*_MS_ = 1.122, *SD* = 0.134) and DPBS + Bacterin group (*K*_PBS+B_ = 1.095, *SD* = 0.120) indicating maintenance of body condition across treatment groups during the study (Figure 2a).

A two-way ANOVA was conducted to evaluate the impact of vaccine formulation and sampling time point on the specific growth rate (SGR, %*G*) of the vaccinated Atlantic salmon parr. No statistically significant interaction between formulation and time point was observed on %*G* (*F*(5, 24) = 0.2212, *p* = 0.9499). Significant main effects of formulation (*F*(5, 24) = 2.761, *p* = 0.0416) and time point (*F*(1, 24) = 9.206, *p* = 0.0057) were detected. The sentinel and DPBS + Bacterin group were excluded from the analysis, however fish demonstrated positive growth throughout the study and are shown in Figure 2b. No significant differences were observed between treatment groups post-injection by Tukey’s HSD post hoc analysis, although the omnibus model detected significant main effects of treatment and time point (Figure 2b).

### 3.3. Macroscopic and Microscopic Pathology Indicate Formulation-Associated Side Effects

#### 3.3.1. External Gross Pathology

**Figure 3:**
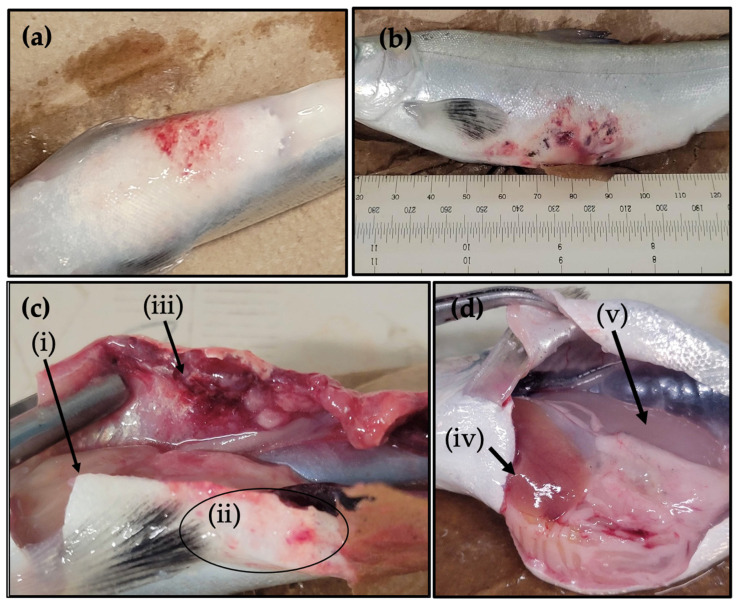
Representative external and internal gross pathology in Atlantic salmon following vaccination with amidated TO-CNF formulations. (**a**,**b**) External multifocal reddening/erythema of the ventral body wall surrounding injection sites in fish vaccinated with (**a**) 2× EDC TO-CNF and (**b**) 2× ODA TO-CNF. (**c**) Internal gross lesions observed in fish that died or were euthanized due to morbidity following 2× EDC TO-CNF vaccination, including (i) pale/mottled liver, (ii) external multifocal erythematous tissue surrounding the injection site, and (iii) proliferative and hemorrhagic tissue extending from the injection site through the body wall into the coelom with adhesions to the pyloric caeca. (**d**) Internal gross findings observed in surviving fish at the 300-degree day sampling time point following vaccination with 2× TO-CNF formulations, including (iv) pale liver and (v) edema within the stomach wall. Representative images are shown; lesion prevalence in surviving fish is summarized in Supplemental Table S1.

Among mortalities, external white fungal growth consistent with *Saprolegnia* sp. (Saprolegniosis) was observed in ten of the twenty-five fish (40%), exclusively in the 2× ODA and 2× EDC TO-CNF treatment groups. Multifocal reddening/erythema of the ventral surface surrounding the injection site was observed in nine of the twenty-five mortalities (36%), also confined to the 2× ODA and 2× EDC TO-CNF treatment groups (Figure 3a,b).

In surviving fish at scheduled time points, the prevalence of external proliferative masses and lesions is summarized in Supplemental Table S1. Briefly, at 300-degree days post-injection sampling, small external proliferative masses surrounding the injection sites were noted from several treatment groups. By 600-degree days, these proliferative masses appeared to progress to ulcerative lesions in a subset of fish vaccinated with amidated TO-CNF formulations (Supplemental Table S1). No comparable lesions were observed in sentinel fish or sham-injected controls.

#### 3.3.2. Internal Gross Pathology

Internal gross examination of mortalities revealed mottled or pale livers (36.0%) and multinodular, proliferative, and erythematous tissue (granulomas) at injection sites extending through the body wall and into the coelomic cavity, frequently accompanied by visceral adhesions involving the intestinal tract and mesentery at the pyloric caeca. These lesions were most frequently observed in fish vaccinated with 2× ODA and 2× EDC TO-CNF groups (40.0%; Figure 3c).

In surviving fish at scheduled sampling time points, internal gross pathology included severe lesions, ascites, pale liver, and gastric wall edema, was frequently noted at the 300-degree day sampling time point only in fish vaccinated with the 2× reagent-loaded TO-CNF formulations but was not observed at the 600-degree day post-injection sampling time point (Figure 3d).

**Figure 4:**
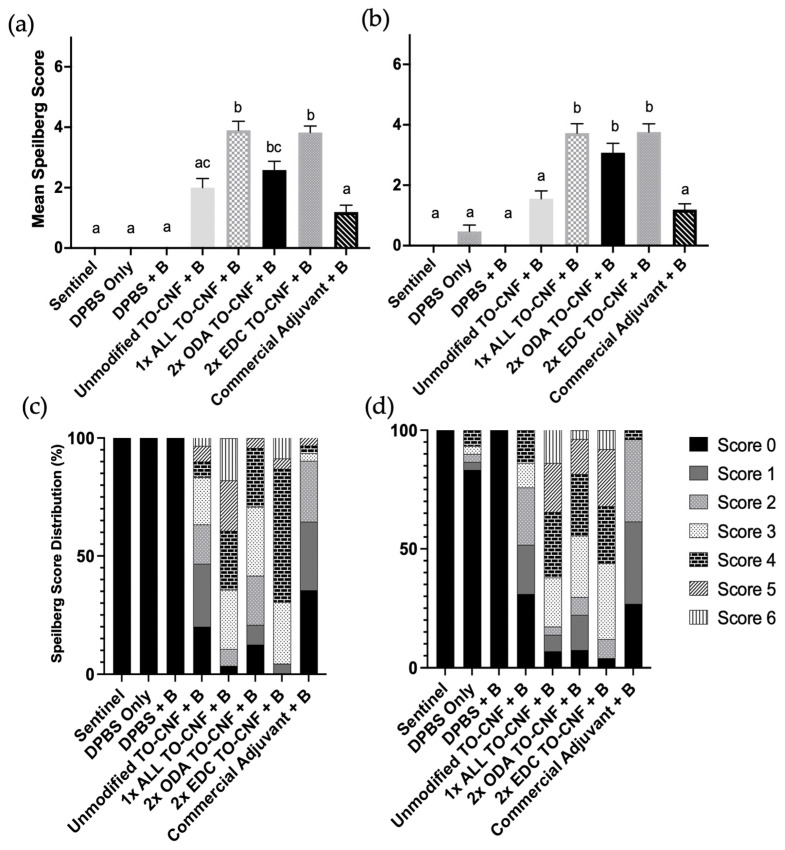
Effect of vaccination formulation on abdominal adhesions in Atlantic salmon as determined by Spielberg scoring rubric at (**a**) 300-degree days and (**b**) 600-degree days post vaccination. Different letters indicate statistically significant differences between treatment groups (Kruskal–Wallis with Dunn’s post hoc test; *p* < 0.05). Distribution of Spielberg scores (%) for internal abdominal adhesions seen in Atlantic salmon peritoneum at (**c**) 300-degree days and (**d**) 600-degree days post-injection with TO-CNF hydrogels. Error bars represent standard error of the mean (*n* = 30).

Internal gross pathology at 300- and 600-degree days post-injection was quantified using the modified Spielberg rubric. A non-parametric Kruskal–Wallis ANOVA revealed significant treatment effects on adhesion scores at both 300-degree days (*H*(8) = 111.7, *p* < 0.0001; Figure 4a) and 600-degree days post-injection (*H*(8) = 98.5, *p* < 0.0001; Figure 4b). Pairwise comparisons using Dunn’s post hoc test identified statistically significant differences in mean adhesion scores between treatment groups, denoted by differing letters in Figure 4a,b. Visceral and peritoneal melanization was observed only in fish vaccinated with 1× ALL, 2× ODA, and 2× EDC TO-CNF formulations at 600-degree days post-vaccination; however, these findings were generally mild and infrequent.

#### 3.3.3. Comparative Histopathological Scoring of Body Wall and Coelom

**Figure 5:**
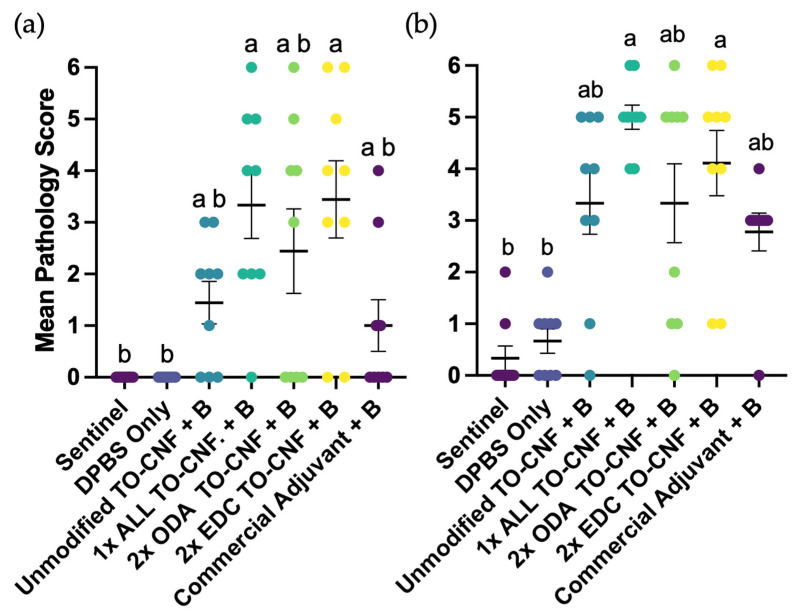
Histopathology scores from (**a**) the body wall and (**b**) the coelom of Atlantic salmon at 600-degree days post-injection. Dots represent raw individual scores and horizontal lines represent mean pathology scores. Different letters indicate statistically significant differences between treatment groups (Kruskal–Wallis with Dunn’s post hoc test; *p* < 0.05). Error bars represent standard error of the mean (*n* = 9).

Microscopic lesions, including fibrosis and inflammation in the body wall and coelom, were described and scored, then analyzed by non-parametric Kruskal–Wallis ANOVA. Statistical differences in mean body wall pathology scores were observed among treatment groups (*H*(7) = 27.64, *p* = 0.0001; Figure 5a). Mean coelom pathology scores also differed significantly across formulations (*H*(7) = 35.44, *p* < 0.0001; Figure 5b) Pairwise comparisons using Dunn’s post hoc analysis identified significant differences between treatment groups, as indicated by letter groupings in Figure 5a,b.

#### 3.3.4. Exploratory Relative Expression of Immunoglobulin Genes Following Vaccination

**Figure 6:**
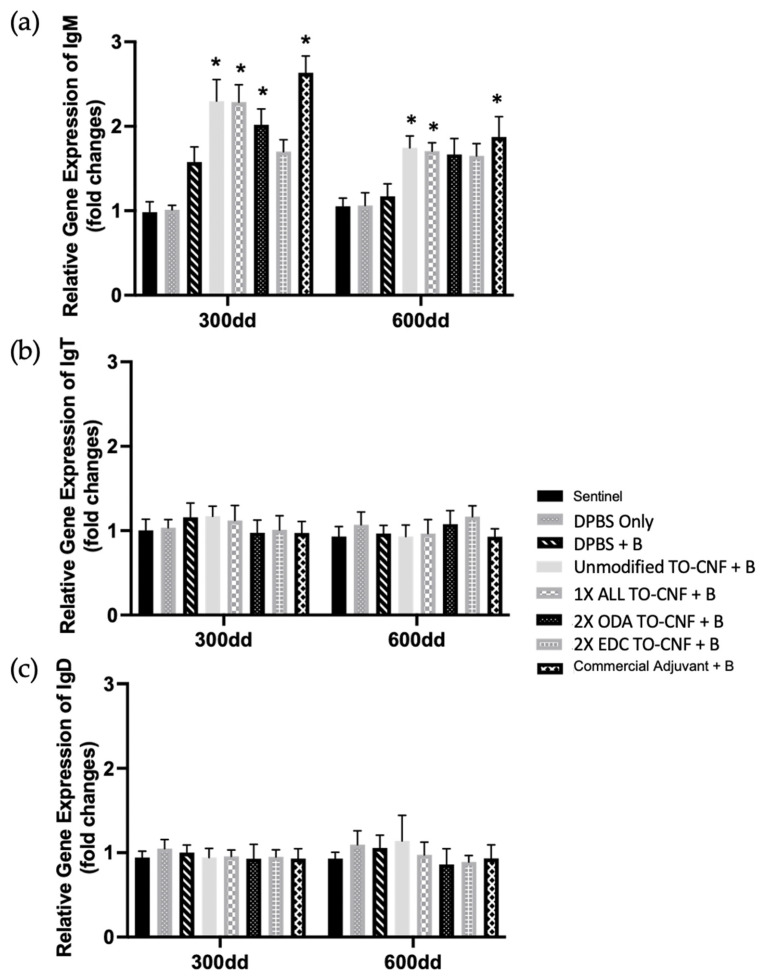
Effects of vaccine formulation on mean gene expression from fish head kidney at 300-degree days and 600-degree days post-vaccination. The 2^−ΔΔCt^ method was used to compare the expression of (**a**) IgM (* denotes treatments where *p* < 0.05 compared to DPBS Only group at each timepoint) (**b**) IgT (**c**) IgD relative to that of *β-actin*. Sentinel and DPBS + Bacterin groups were not included in statistical analysis. Error bars represent standard error of the mean (*n* = 9).

Exploratory relative expression of immunoglobulin genes (*IgM*, *IgD*, and *IgT*) in the head kidney of vaccinated Atlantic salmon was measured to assess immunological responses to amidated TO-CNF formulations and compared to those receiving the commercial oil-adjuvanted vaccine. Gene expression was normalized to the reference gene, *β-actin*. Sentinel unvaccinated fish and DPBS + Bacterin controls were excluded from statistical analysis due to unequal sample sizes; however, their expression profiles were comparable to those of analyzed treatment groups and shown (Figure 6). Ordinary two-way ANOVA revealed significant main effects of time and treatment on IgM expression, with no significant time x treatment interaction. No significant effects of treatment or time were detected for *IgD* and *IgT* expression. Where overall treatment effects were detected, Dunnett’s post hoc test identified formulations with relative expression levels differing significantly (*) from the DPBS-only sham control at the corresponding time point (Figure 6a). *IgT* and *IgD* expression remained unchanged across treatments and time points (Figure 6b,c).

#### 3.3.5. Serological Antigen-Specific Antibody Response by Indirect ELISA

**Figure 7:**
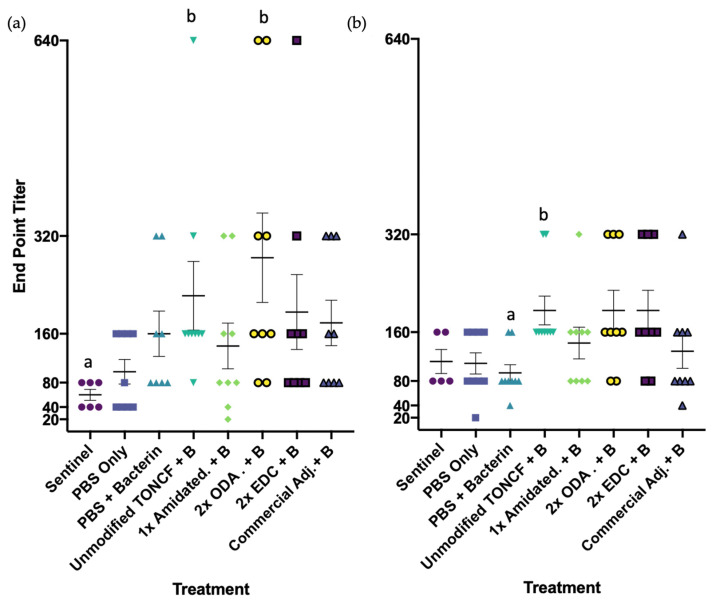
Antigen-specific IgM antibody endpoint titers in vaccinated Atlantic salmon serum at (**a**) 300-degree days (*p* = 0.0129) and (**b**) 600-degree days post-vaccination (*p* = 0.0109). Dots represent raw individual titers and horizontal lines represent group means. Different letters indicate statistically significant differences among treatment groups (Kruskal–Wallis with Dunn’s post hoc test; *p* < 0.05). Error bars represent standard error of the mean (*n* = 9).

Exploratory indirect ELISA was used to quantify endpoint titers of antigen-specific IgM in serum from the same fish analyzed by real time qPCR, allowing comparison of IgM transcriptional responses corresponded to *Vibrio anguillarum*-specific humoral antibody responses. Mean intra-assay and inter-assay coefficients of variation for assay validation were calculated to be 8.53 ± 2.75% (duplicate wells) and 11.62 ± 3.25% (plate-to-plate), respectively, consistent with acceptable ELISA performance. Mean endpoint titers were analyzed using non-parametric Kruskal–Wallis ANOVA. Statistical differences among treatment groups were observed at both 300-degree days (*H*(8) = 17.79, *p* = 0.0129; Figure 7a). and 600-degree days post-vaccination (*H*(8) = 18.24, *p* = 0.0109; Figure 7b). Pairwise comparisons using Dunn’s post hoc test identified statistically significant differences between treatment groups, as indicated by letter groupings (Figure 7a,b).

Because these immune endpoints were exploratory and antigen-specific responses showed variability, these results should be interpreted cautiously as indicators of immune activation rather than definitive evidence of adjuvant activity.

### 3.4. Physicochemical Characterization Indicates Differences in Structural Behavior and Potential Chemical Residuals in Amidated TO-CNF Formulations

#### 3.4.1. Shear-Thinning Flow Behavior of TO-CNF Formulations by Rheology

Rheology analysis was used to characterize flow behavior of the amidated TO-CNF hydrogel formulations under applied shear with friction maintained at max shear rate tested (*γ* = 10 s^−1^). It is important to note that oscillatory moduli (G′/G″) were not measured; rheological analysis was used primarily to characterize injectability-relevant flow behavior. Formulations with increased reagent loading (2× EDC and 2× ODA) exhibited sample failure at lower shear rates (*γ* = 3 s^−1^), characterized by either sample slipping or fracture, and could not be analyzed for rheological properties.

**Figure 8:**
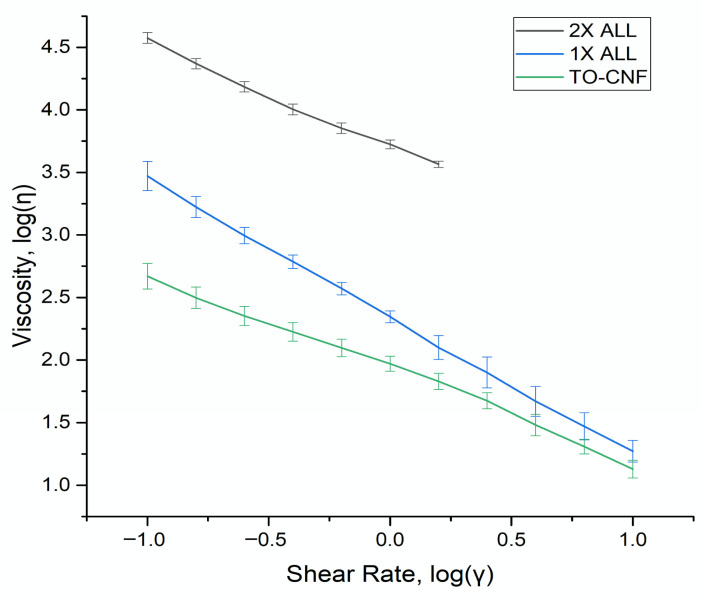
Log-log plot of mean viscosity (*η*) in relation to mean shear rate (*γ*) for shear-thinning TO-CNF formulations exhibiting power-law behavior (*n* = 3). Error bars are standard deviations from the mean.

Viscosity (*η*) and shear rates (*γ*) were logarithmically graphed from amidated TO-CNF formulations that did not exhibit sample failure. As shear rate increased, viscosity decreased, with all tested formulations exhibiting a negative linear slope consistent with shear-thinning, power-law fluid behavior. No plateau or positive linear slope regions were observed, indicating an absence of Newtonian and shear-thickening behavior within the tested range. Representative results are shown in Figure 8.

The flow behavior index was calculated according to Tanner 2000 with values expected to increase with increased network interactions or structural organization [50]. All samples demonstrated shear-thinning characteristics as indicated by the negative power-law slopes and flow behavior indices. Unmodified TO-CNF exhibited a power-law slope of −0.95923 and a flow behavior index of 0.0334. The 1× ALL formulation showed a power-law slope of −0.88028 and a flow behavior index of 0.0493, indicating stronger shear-thinning behavior relative to the unmodified material. The 2× ALL formulation exhibited a power-law slope of −0.83082 and a flow behavior index of 0.1776. Contrary to expectations, increasing reagent loading did not produce a monotonic change in flow behavior consistent with cross-linking behavior, with the most pronounced shear-thinning behavior observed in the 1× ALL samples, as indicated by the largest overall change in viscosity with increasing shear rate. 

#### 3.4.2. Structural Differences in TO-CNF Formulations by SEM

Scanning electron microscopy (SEM) was used to visualize cross sections of each TO-CNF formulation for surface properties and homogeneity. The SEM images for each TO-CNF formulation type had observed differences. The 2× ALL TO-CNF formulations showed visible high-level of branching and unorganized flaking under 250× magnification. Under 1500× and 2500× magnification, this branching appeared as webbing-like structures. The 1× ALL TO-CNF formulation demonstrated porous surface with organized flakes and slight clumping under 250× magnification and under 1500× magnification, this was described as flakes with mild webbing present. Amidated TO-CNF formulations appeared distinct to unmodified TO-CNF formulations at 250× and at 1500× with organized flakes and no branching, clumping, nor webbing observed (Supplemental Figure S1).

#### 3.4.3. Qualitative FT-IR Assessment of Chemical Features in TO-CNF Formulations

**Figure 9:**
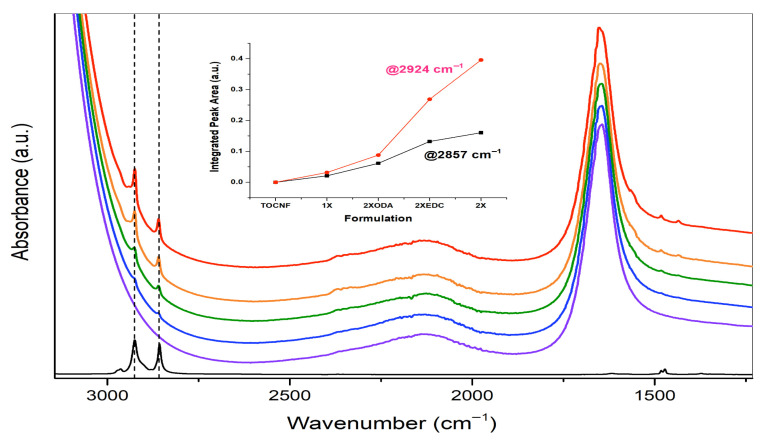
FT-IR interferogram documenting changes in absorbance across variations in TO-CNF formulations (Red: 2× ALL TO-CNF; Orange: 2× EDC TO-CNF; Green: 2× ODA TO-CNF; Blue: 1× ALL TO-CNF; Purple: Unmodified TO-CNF; Black: Octadecylamine) of a zoomed in area of the absorbance spectrum between wavelengths 3300 to 1200 cm^−1^. Subset graph shows the integrated peak area of each formulation at 2857 and 2924 cm^−1^ (dotted vertical lines on spectrum).

Because rheology and SEM results suggested differences in structural uniformity among formulations, ATR-FT-IR was used to present integrated absorbance for relative comparison across formulations measured under identical conditions. This was used to qualitatively assess spectral differences among formulations and evaluate features consistent with chemical modification or the presence of features consistent with residual or trace components remaining after production. First, amidated TO-CNF formulations were analyzed in comparison to unmodified TO-CNF by FT-IR (Figure 9).

Within all variations in the amidated TEMPO CNF there were twin peaks present at the wavelength of 2924 cm^−1^ and 2857 cm^−1^. These twin peaks are consistent with the presence of alkyl (-CH_2_) groups along the ODA saturated-hydrocarbon chain suggesting successful ODA conjugation. Absorbance peaks at these locations increased with reagent loading conditions, as reflected by integrated absorbance (semi-quantitative comparison only). Also noteworthy, twin absorbance peaks at wavelength 1450 cm^−1^ were visible with increasing ODA/DMF and EDC/NHS concentrations (Figure 9).

Further, FT-IR was used to qualitatively examine the signals consistent with compositional differences following sequential wash steps during the hydrogel formulation process. Supplemental Figure S2 suggests that absorbance peaks associated with EDC/NHS and HCl were reduced or no longer detectable, however features consistent with ODA/DMF-related components and ethanol diminish but do not disappear across washes. These observations, in conjunction with the adverse events observed *in vivo*, suggest that formulation composition and purification may influence the safety outcomes; however, this cannot be confirmed nor attributed to a specific driver with the current data, and warrants further investigation of purification strategies and/or alternative chemistries (Supplemental Figure S2).

## 4. Discussion

Injectable biopolymer hydrogels have gained increasing attention as delivery systems for vaccines and therapeutics [51] due to their ability to combine minimally invasive administration with the functional advantages of three-dimensional crosslinked matrices, including high cargo loading capacity, tunable mechanical properties, and prolonged residence at the site of administration [52]. These systems also leverage polymer materials that are readily available, environmentally sustainable, and cost-effective [53]. In terrestrial and human medicine, shear-thinning and injectable hydrogels have shown promise as vaccine adjuvant and drug delivery systems [44]. However, their biological performance and safety profiles remain poorly characterized in finfish, where intraperitoneal vaccination is routine and material-induced inflammatory responses can have significant consequences for animal welfare and production sustainability [9,10,11,12,13,14,15]. The present study was therefore designed as an *in vivo* safety-focused evaluation of injectable amidated TEMPO-oxidized cellulose nanofiber (TO-CNF) hydrogels formulated with a *Vibrio anguillarum* bacterin in Atlantic salmon.

Amidation chemistry was employed to modify TO-CNF in order to promote hydrogelation through hydrophobic interactions and hydrogen bonding [54], yielding shear-thinning formulations capable of administration through a small-gauge needle. This approach was selected to reduce the invasiveness associated with implanted hydrogels and to mitigate foreign body responses previously observed with citric-acid crosslinked TO-CNF materials [27]. Similar strategies have been explored in other biopolymer systems such as Chiu et al., 2009 where a molecularly branched aqueous form of hydrogelating chitosan demonstrated acceptable biocompatibility and macrophage activation in mammalian models [29].

However, amidation represents a substantial chemical modification of the CNF surface and requires the use of reactive reagents including octadecylamine (ODA), N,N-dimethylformamide (DMF), and carbodiimide coupling agents, all of which are known biological hazards [55,56,57]. ODA is listed in the PubChem Compound Summary as a corrosive irritant capable of negatively impacting skin, eyes, and mucous membranes. It is used commonly for emulsions and as a viscosity controlling agent including as a stabilizer in cosmetics [55]. Furthermore, the LD_50_ for ODA has been quantified previously as 250 mg/kg delivered intraperitoneally by injection in mice [56]. With respect to N,N-dimethylformamide (DMF), US-EPA examination in fish showed the acute toxicity of DMF to three freshwater fish species ranging from EC/LC_50_ (96 h) = 7100–10,600 mg/L [57]. Consequently, extensive washing and dialysis were implemented to remove residual reaction components. Nonetheless, the potential biological consequences of both the concentrations of residual reaction components and the amidated TO-CNF chemistry itself following intraperitoneal administration in fish were unknown at the outset of the study. These residual reaction components were not quantitatively measured in this study, and therefore their contribution to observed biological effects documented cannot be definitively distinguished from other formulation characteristics.

To develop the shear-thinning CNM hydrogel as a potential vaccine technology, TO-CNF was chosen as the target nanomaterial formulated with a *Vibrio anguillarum* bacterin. This global pathogen was used to produce a whole-cell inactivated bacterin acting as the antigen due to being well studied, characterized, and widely used in commercial oil-adjuvanted vaccines for Atlantic salmon and other aquacultured species [58]. The *Vibrio anguillarum* bacterin was mixed after production with the TO-CNF formulations at a ratio similar to the current oil-based injection vaccine formulations.

The *in vivo* trial revealed pronounced, reagent-loading-dependent adverse outcomes associated with the higher-modified amidated TO-CNF formulations. Fish vaccinated with both 2× reagent-loaded formulations exhibited significantly increased mortality by 300-degree days post-injection relative to both sham-injected controls and a commercial oil-adjuvanted vaccine. A sentinel unvaccinated control group maintained separately did not exhibit macroscopic nor microscopic pathology, nor morbidity or mortality supporting that observed outcomes were treatment-associated rather than due to environmental or infectious disease. Furthermore, the inclusion of sentinel fish injected with DPBS + Bacterin as a post hoc control supports that the inactivated *Vibrio anguillarum* antigen alone was not responsible for the observed adverse events. Hazard ratio analysis further demonstrated elevated mortality risk in fish receiving 2× reagent-loaded TO-CNF formulations, while fish vaccinated with unmodified TO-CNF or 1× ALL TO-CNF formulations did not differ significantly from the commercial oil-adjuvanted vaccine. These findings identify modification-level-dependent safety limitations associated with the tested amidated TO-CNF hydrogels in Atlantic salmon. Because multiple formulation properties (including degree of modification, hydrophobicity, and potential residual components) varied simultaneously across formulations, these findings should be interpreted at the formulation level rather than as evidence of any single mechanistic or physicochemical factor.

Biometric data were collected to monitor growth. This was first accomplished by calculating the coefficient of condition (*K*). Fulton’s condition factor expresses the relative robustness, or degree of well-being, of a fish. Variations in *K* are associated with the degree of nourishment and general health of the fish. Condition values may also fluctuate with fish age, stage of reproductive maturity, and in some species, with sex [59]. Intraperitoneal injections of amidated TO-CNF formulations, particularly the 2× modifications, into Atlantic salmon parr were associated with observable adverse effects including reduced observed feeding. However, vaccine formulation showed no significant impact on Fulton’s condition factor (*K*_mean_ ≥ 1.0) compared to baseline or sham-injected controls, suggesting this metric lacks the sensitivity needed to detect adverse impacts on growth during short duration vaccine safety and efficacy studies. For this reason, post hoc specific growth rate (SGR) was calculated and analyzed. SGR expresses growth as the percent change in size per unit time [60]. While ordinary two-way ANOVA detected significant effects of time and treatment, Tukey’s HSD post hoc analysis revealed no significant pairwise differences among treatments. Accordingly, these results do not support treatment-specific effects on growth and should be interpreted cautiously, as the observed model-level significance does not translate to biologically meaningful differences between formulations.

The modified Spielberg adhesion scoring system provides a semi-quantitative assessment of adhesions, melanization, and residuals with increasing scores reflecting greater severity of fibrous adhesion formation and associated inflammatory response. This scoring system has been widely used in fish vaccine studies and has been shown to generally correlate with underlying histopathological changes, supporting its relevance as a biologically meaningful indicator of local tissue response [9,10,11,12]. Macroscopic and microscopic pathological assessments revealed pale livers, ascites, edema within the stomach, localized inflammatory responses, visceral adhesions, fibrosis, and granuloma formation in fish vaccinated with amidated TO-CNF formulations, with severity increasing at higher modification levels compared to negative sham injected controls, unmodified TO-CNF, and the commercial oil-adjuvant vaccine. During external examination, signs of infection consistent with the eukaryotic freshwater fish pathogen, *Saprolegnia*, were observed in a subset of fish, particularly among individuals that experienced severe adverse reactions. Saprolegnia infections are well documented to occur in association with inflammation and chronic stress-induced immunosuppression in salmonids [61]. Loss of mucosal immune defenses in the skin and/or gill tissue facilitates fungal invasion [61,62]. *Saprolegnia*-like growth was observed only in a subset of severely affected fish and is interpreted as a secondary opportunistic finding, as such infections are well documented in compromised individuals and were not observed in control or lower-modification groups. This pattern is consistent with these lesions reflecting underlying host compromise rather than a direct treatment effect.

Exploratory immune analyses indicated variable gene expression responses following vaccination with unmodified and 1× ALL TO-CNF formulations, as evidenced by detectable differences in *IgM* heavy chain gene expression in some treatment groups, although responses were variable and should be interpreted cautiously as exploratory. No significant differences were observed in expression of *IgD* nor *IgT*, which is expected as these two antibodies occur at much lower concentrations than IgM and are specialized to the gut, gills, and spleen (*IgD*), and mucosal immunity (*IgT*) [63,64]. However, antigen-specific *IgM* titers measured by ELISA were highly variable and did not differ significantly among vaccinated groups. Previous research has demonstrated Atlantic salmon receiving a commercial oil-adjuvanted vaccine stimulate antibody production and have IgM concentrations two to four times as high compared to unvaccinated fish [65,66]. Importantly, antibody levels alone are often not reliable predictors of protective immunity in salmonids [66,67,68], and no challenge study was conducted to assess vaccine efficacy due to the adverse effects observed. Moreover, the antibody responses observed in higher-dose amidated formulations raise the possibility that immune activation may, in part, reflect nonspecific inflammation rather than controlled vaccine-mediated immunity. As such, the immunological findings should be interpreted cautiously and viewed as exploratory rather than indicative of protective efficacy.

Physicochemical characterization performed in parallel with the *in vivo* study provided contextual information on formulation chemistry, structure, and behavior. Rheological analyses provided useful comparative insights into formulation handling and shear-thinning behavior, and confirmed enhanced shear-thinning behavior in amidated TO-CNF formulations; however, higher-modification hydrogels (2× EDC and 2× ODA) exhibited mechanical instability, including sample fracture and slippage, consistent with differences in material structure observed with SEM and FT-IR results [69], and were not used to infer direct relationships with *in vivo* outcomes. Scanning electron microscopy was used to qualitatively assess material homogeneity and revealed increased fibril aggregation and branching in 2× reagent-loaded formulations, but these structural observations were not used to directly predict biological performance. FT-IR analysis provided spectral features consistent with ODA modification but also provided qualitative spectral evidence that may indicate the possibility of trace residual reaction components following washing and dialysis. Importantly, FT-IR does not quantify residual reagent concentrations [70], and these data did not permit definitive attribution of toxicity to residual chemicals versus the intrinsic effects of the amidated TO-CNF itself, particularly at higher degrees of modification. Accordingly, FT-IR results were interpreted only as qualitative compositional context rather than as evidence of causation. Both residual reagent toxicity and intrinsic chemistry-dependent inflammatory responses represent plausible but unconfirmed risk profiles for injectable adjuvants, irrespective of their relative contribution. Chromatographic quantification of residual reagents was not performed, as the pronounced adverse *in vivo* effects observed did not justify additional mechanistic resolution within the scope of this safety-focused investigation but could be a focus area of future work. Taken together, these results demonstrate that increasing reagent loading and degree of chemical modification are associated with reduced survival and increased pathological responses. However, the specific mechanisms underlying these effects, including potential contributions from residual reaction components, physicochemical instability, or purification efficiency, cannot be determined from the present study and remain inferential.

## 5. Conclusions

From an applied aquaculture perspective, the safety profile observed for amidated TO-CNF hydrogels, particularly at higher modification levels, precludes their immediate suitability for commercial vaccine applications. Injectable materials that induce significant inflammation, adhesions, or mortality are unacceptable with animal welfare standards, regulatory requirements, and sustainable production practices. These findings do not support the use of these formulations as a viable vaccine delivery platform and identify critical safety constraints associated with the tested amidated TO-CNF formulations in finfish. These findings are based on observed associations between formulation conditions and biological outcomes, and do not establish specific mechanistic drivers of toxicity.

Importantly, the findings provide valuable guidance for future materials-focused vaccine research. Unmodified TO-CNF and lower-modification formulations demonstrated improved tolerability and limited and variable evidence of immune activation without severe pathology, suggesting that CNF-based systems warrant further investigation when hazardous chemistries are avoided. These findings define safety constraints for the tested formulations, indicating that the amidated TO-CNF hydrogels examined here are not suitable under the conditions tested for use as injectable vaccine adjuvants in Atlantic salmon, but do not preclude the development of safer cellulose-based systems using alternative chemistries. Future work should prioritize “green” modification strategies, physical or ionic crosslinking approaches, or unmodified CNM formulations to achieve injectability and immune modulation while minimizing adverse biological responses. More broadly, this study calls attention to the importance and necessity of rigorous *in vivo* safety evaluation early in the development of injectable biomaterial-based vaccines prior to efficacy examination for aquaculture species.

## 6. Limitations of the Study

One of the most significant limitations of this study was the pronounced adverse impacts observed *in vivo*, particularly in fish injected with higher-concentration (2×) amidated TO-CNF formulations. Despite rigorous washing and dialysis, Fourier transform infrared spectroscopy (FT-IR) provided qualitative spectral features consistent with the possible presence of trace reaction components, such as ODA, DMF, and/or ethanol, in the final formulations. Residual components were not quantitatively measured; therefore, definitive attribution of the observed tissue pathology to residual reagents versus intrinsic effects of the chemically modified TO-CNF itself cannot be made. The external and internal inflammation, visceral adhesions, secondary fungal infections, and elevated mortality rates observed therefore may be associated with a combination of material properties and formulation-related factors; however, the relative contribution of potential residual components cannot be determined from the present data.

These adverse outcomes highlight important safety concerns regarding the safety of chemically modified TO-CNF hydrogels when reactive reagents are employed. From an applied aquaculture perspective, these formulations, which were associated with severe inflammatory responses or mortality, would not be acceptable from regulatory, welfare, and environmental perspectives, which would limit scalability and commercial acceptance irrespective of formulation performance.

Histopathological evaluation did not include the DPBS + Bacterin control group, which limits direct microscopic comparison for antigen-only exposure. Furthermore, because multiple immune parameters were evaluated, these immunological analyses should be interpreted as exploratory and hypothesis-generating rather than confirmatory assessments of adjuvant efficacy, particularly given the small sample size. Immunoglobulin M (*IgM*), while the dominant circulating antibody in teleosts, is not a consistently reliable correlate of protective immunity. The large inter-individual variability observed in antigen-specific *IgM* titers by ELISA, together with the absence of significant differences in *IgD* and *IgT* expression, underscores the need for broader immune profiling and validation through controlled efficacy trials. However, vaccine challenge studies were not pursued in the present work due to the severity of adverse events observed in some treatment groups, precluding scientifically and ethically justifiable continuation of efficacy testing. In addition, the study duration (600 degree-days) and addition of a post hoc control may not have been sufficient to capture longer-term effects on immune function, growth performance, or chronic pathology.

Finally, the physical properties of the hydrogel formulations presented additional limitations. Non-uniform material composition and mechanical instability, observed through rheological testing and scanning electron microscopy, suggesting that increased reagent concentrations may be associated with reduced hydrogel homogeneity and structural integrity. Formulations differed in multiple physicochemical properties (e.g., degree of modification, hydrophobicity, and potential residual components), which limits the ability to isolate individual causal drivers. Collectively, these limitations emphasize the need for alternative formulation strategies that prioritize material uniformity, quantitative assessment, biocompatibility, and predictability. Future work should focus on safer, “green” modification approaches or unmodified CNM systems to better define conditions under which shear-thinning CNM-based hydrogels may be evaluated responsibly for aquaculture vaccine applications.

## Figures and Tables

**Table 1:** Summary of TO-CNF formulations in the experimental design.

Formulation Name	TO-CNF Modification	Modification Difference	Sonication	Purification	Antigen (Bacterin)	Intended Role
Sham (DPBS)	None	N/A	No	None	No	Negative control
DPBS + Bacterin	None	N/A	No	None	Yes	Antigen control
Unmodified TO-CNF + Bacterin	Unmodified	N/A	No	None	Yes	Material control
1× ALL TO-CNF + Bacterin	Amidation	Baseline reagent loading (1×)	Yes	Washed and Dialyzed	Yes	Baseline amidation
2× ODA TO-CNF + Bacterin	Amidation	Increased ODA + DMF (2×)	Yes	Washed and Dialyzed	Yes	Increased hydrophobicity
2× EDC TO-CNF + Bacterin	Amidation	Increased EDC + NHS (2×)	Yes	Washed and Dialyzed	Yes	Increased coupling density
Commercial oil-adjuvanted vaccine	None	N/A	No	None	Yes	Positive control

**Table 2:** Primer sequences used in real-time qPCR for the determination of gene expression.

Genes	Component	Sequences (5′–3′)	Gene Accession No	Product Size	Reference
*IgM*	Forward Reverse	AGGCGGAAATTCCCTGACTG CACGGAGTTGACTGACTCCC	Y12457.1	83	(Habte-Tsion et al., 2024) [45]
*IgD*	Forward Reverse	CGTCTACTCCATCGCTCCAC TTTGGCGTCATACGCAGAGT	AF141607.1	104	(Habte-Tsion et al., 2024) [45]
*IgT*	Forward Reverse	CAAAGGGCAACCTGAACAGC GAACGACCGGTGTGTCTTCA	GQ907004.1	117	(Habte-Tsion et al., 2024) [45]
*β-actin*	Forward Reverse	CCAAAGCCAACAGGGAGAA AGGGACAACACTGCCTGGAT	BG933897	102	(Olsvik et al., 2011) [47]

Note: *IgM*, immunoglobulin M; *IgD*, immunoglobulin D; *IgT*, immunoglobulin T; *β-actin*, beta-actin (reference gene).

**Table 3:** Mantel-Haenszel hazard ratios of mortality in Atlantic salmon vaccinated with formulations compared to the commercial oil-adjuvanted vaccine group (Referent) at 600-degree days post-injection using Gehan–Breslow–Wilcoxon analysis.

Formulation	% Cumulative Incidence (No. Morts/No. Total)	% Cumulative Survival (No. Censored/No. Total)	M-H Hazard Ratio (95% CI) *x*^2^ (df), *p*-Value
Sentinel (Unvaccinated)	0 (0/120)	100 (120/120)	0.0498 (0.00078–3.183) 2.000 (1), *p* = 0.3173
DPBS Only (Sham Negative Control)	0 (0/60)	100 (60/60)	0.1353 (0.0027–6.821) 1.000 (1), *p* = 0.3173
DPBS + Bacterin (Antigen Control)	0 (0/20)	100 (20/20)	0.2636 (0.0029–24.36) 0.333 (1), *p* = 0.5637
Unmodified TO-CNF + Bacterin (Material Control)	0 (0/60)	100 (60/60)	0.1353 (0.0027–6.821) 1.000 (1), *p* = 0.3173
1× ALL TO-CNF + Bacterin	5.0 (3/60)	95 (57/60)	2.719 (0.383–19.300) 0.9748 (1) *p* = 0.3235
2× ODA TO-CNF + Bacterin	16.7 (10/60)	83.3 (50/60)	5.461 (1.670–17.860) 7.728 (1) ***p* = 0.0054**
2× EDC TO-CNF + Bacterin	18.3 (11/60)	81.7 (49/60)	5.713 (1.830–17.830) 8.828 (1) ***p* = 0.0030**
Commercial oil-adjuvanted vaccine (Positive Control)	1.7 (1/60)	98.3 (59/60)	Referent

Note: M-H: Mantel-Haenszel hazard ratio; CI: 95% confidence interval. Value in bold denotes statistically significant differences between mean survival curves in formulations that were intraperitoneally injected Atlantic salmon compared to commercial oil-adjuvanted vaccine (*α* = 0.05).

## Data Availability

The data presented in this study are available from the corresponding author upon reasonable request.

## Supplementary Materials

The following supporting information can be downloaded at: https://www.mdpi.com/article/10.3390/vaccines14040313/s1. Table S1: Prevalence of external proliferative masses at 300-degree days (dd) and progressive external lesions at 600-degree days surrounding injection site of Atlantic salmon post-injection. Figure S1: Representative SEM photomicrographs of TO-CNF formulations. Figure S2: FT-IR interferograms illustrating removal of unreacted reagents across wash steps following the amidation process.

## Abbreviations

TO-CNF	2,2,6,6-tetramethylpiperidine-1-oxyl radical (TEMPO)-mediated cellulose nanofibers
CNM	cellulose nanomaterials
CNF	cellulose nanofibers
FBR	foreign body response
ELISA	enzyme-linked immunosorbent assay
HR	hazard ratio
CI	confidence interval
M-H	Mantel-Haenszel hazard ratio
B	bacterin
Df	degrees of freedom
DPBS	Dulbecco’s phosphate-buffered saline
ODA	octadecylamine
DMF	dimethylformamide
EDC	-ethyl-3-(3-dimethylaminopropyl) carbodiimide hydrochloride
NHS	N-Hydroxysuccinimide
ANOVA	analysis of variance
SGR	specific growth rate
SS	sum of squares
MS	mean square
SEM	scanning electron microscopy
FT-IR	Fourier transform infrared spectroscopy
