# Supplementary material for: In Vivo Safety Characterization of Injectable Amidated TEMPO-Oxidized Cellulose Nanofiber Hydrogel Vaccine Formulations in Farmed Atlantic Salmon (Salmo salar L.)
Source: Vaccines (Basel). 2026 Mar 31;14(4):313. doi: 10.3390/vaccines14040313 (PMC13120112; doi:10.3390/vaccines14040313)
Supplement: Supplementary file 1 [file vaccines-14-00313-s001.zip › vaccines-4161027-supplementary.pdf]

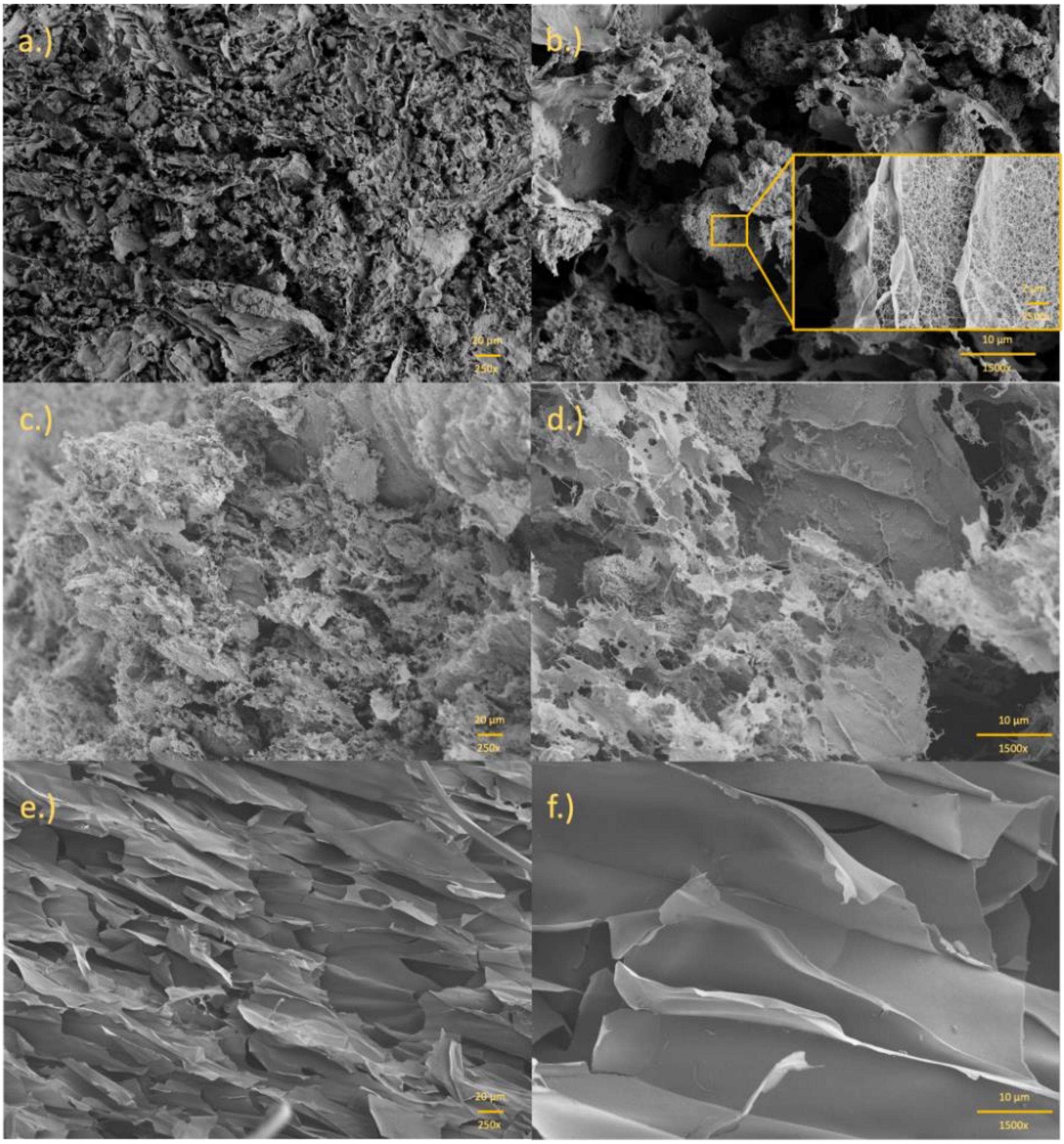

Figure S1. Representative SEM photomicrographs of TO-CNF formulations a.) 2X ALL TO-CNF at 250x magnification b.) 2X ALL TO-CNF at 1500x magnification (with subset magnification at 2500x showing webbing) c.) 1X ALL TO-CNF at 250x magnification d.) 1X ALL TO-CNF at 1500x magnification e.) Unmodified TO-CNF at 250x magnification f.) Unmodified TO-CNF at 1500x magnification Scale bars represent 20 µm under 250x magnification, 10 µm under 1500x magnification, and 2 µm under 2500x magnification.

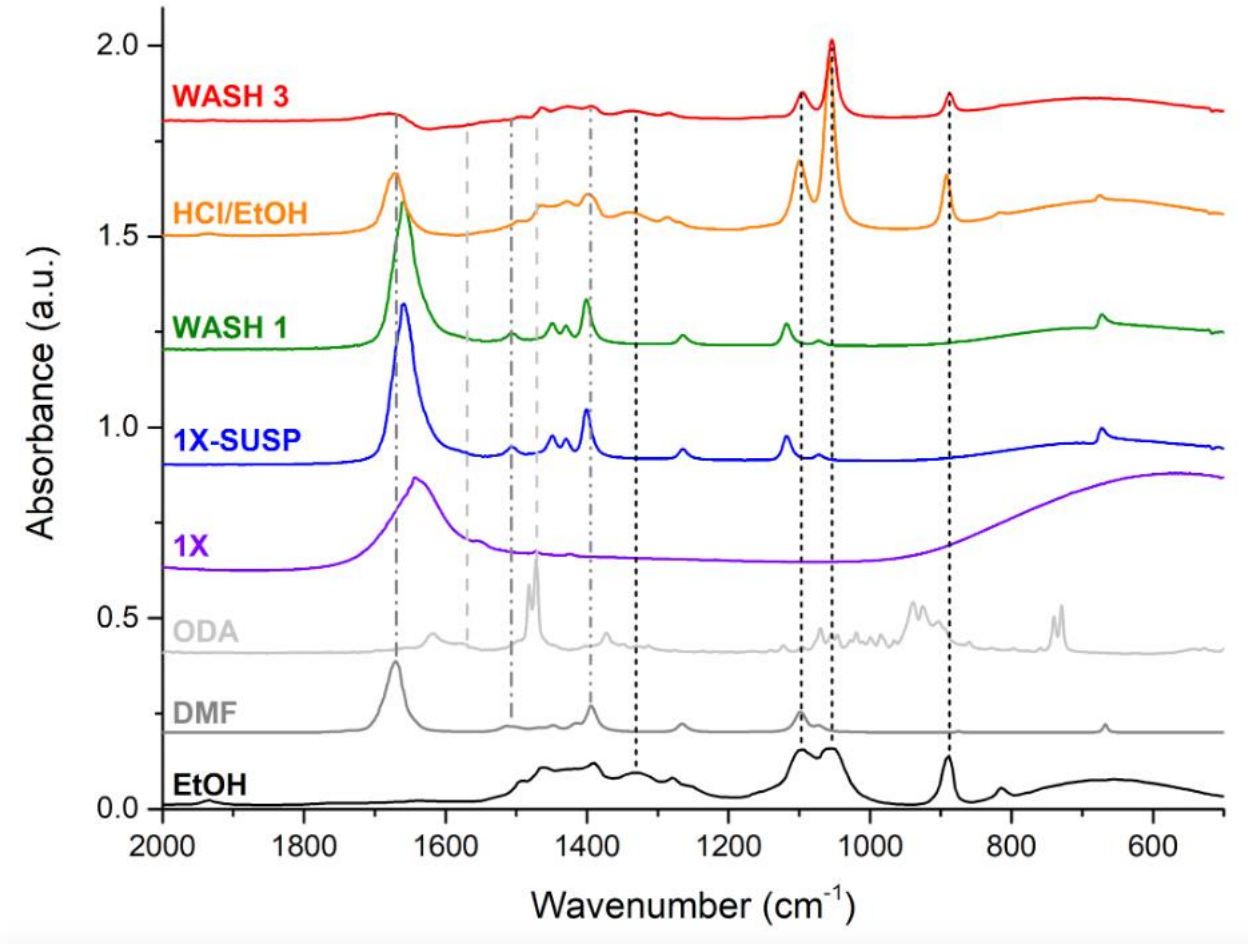

Figure S2. FT-IR interferograms illustrating removal of unreacted reagents following the amidation process. Here, TO-CNF wash supernatants were collected and show progressive reduction of ODA/DMF-associated peaks (dashed vertical lines) across wash steps. Residual peaks after the third wash indicate that unconjugated ODA and DMF were largely eliminated, while some trace EtOH may remain as a result of the HCl/EtOH wash and regeneration step.

Table S1. Prevalence of external proliferative masses at 300- degree days (dd) and progressive external lesions at 600-degree days surrounding injection site of Atlantic salmon post-injection.

	Affected / Total No. Fish (%)	
Treatment Group	300- dd	600- dd
Sentinel (Unvaccinated)	0/30 (0%)	0/30 (0%)
DPBS Only (Sham Negative Control)	0/30 (0%)	0/30 (0%)
DPBS + Bacterin (Antigen Control)	0/10 (0%)	0/10 (0%)
Unmodified TO-CNF + Bacterin (Vehicle Control)	2/30 (6.67%)	0/30 (0%)
1X ALL TO-CNF + Bacterin	3/30 (10%)	4/30 (13.33%)
2X ODA TO-CNF + Bacterin	6/30 (20%)	5/30 (16.67%)
2X EDC TO-CNF + Bacterin	5/30 (16.67%)	5/30 (16.67%)
Commercial Adjuvant + Bacterin (Positive Control)	1/30 (3.33%)	0/30 (0%)
